# Co-Creating Carer-Centric Service Model: Assessing Carer Needs and Developing Needs-Stratified Interventions

**DOI:** 10.1093/geroni/igaf122.031

**Published:** 2025-12-31

**Authors:** Vivian Lou, Rebecca Utz

## Abstract

This symposium aims to explore innovative approaches to enhance the well-being of family carers in Hong Kong through collaborative research and tailored interventions. Utilizing participatory co-creation methods, Jockey Club Carer Space Project underscores the importance of needs-stratified service provision and carer-centric design, illustrating how collaborative research can lead to sustainable, community-driven solutions. Two interventions were developed to support carers of older adults with different levels of need. A randomized controlled trial was conducted to examine effectiveness of a low-intensity cognitive behavioral therapy (CBT)-guided group intervention. Preliminary findings suggest that the intervention may significantly improve mental health outcomes and overall quality of life for family carers of older adults. A two-phase usability evaluation was conducted to collect participants’ feedback on the functionality and user experience of an eCoaching platform, leading to refinements before its launch for evaluating its effectiveness in helping family carers of older adults manage their mental health in a three-arm randomized controlled trial. Quantifying economic burden of family caregiving in Hong Kong, particularly in light of the aging population, highlights the significant monetary value of caregiving time and related out-of-pocket expenses, reiterating the urgent need for policy measures to support family carers in their critical roles. This symposium will provide a platform for discussing innovative, evidence-based interventions that address the complex needs of family carers. By fostering collaboration among researchers, practitioners, and policymakers, this symposium aims to pave the way for more effective carer-centric service model that enhance the quality of life for both carers and care recipients.

